# Spinal dumbbell-shaped epidural cavernous hemangioma (CM): report of nine surgical cases and literature review

**DOI:** 10.1186/s41016-017-0107-2

**Published:** 2018-01-15

**Authors:** Liang Zhang, Zhifeng Zhang, Wuyang Yang, Jifeng Shang, Wenqing Jia, Jun Yang, Yulun Xu

**Affiliations:** 1 Beijing Neurosurgical Institute, Beijing Tiantan Hospital, Capital Medical University, No. 6, Tiantan Xili, Beijing, People’s Republic of China; 2 Department of Neurosurgery, Johns Hopkins University School of Medicine, Baltimore, MD USA

**Keywords:** Cavernous hemangiomas, Spinal cord, Epidural, Dumbbell-shaped, Imaging features, Prognosis

## Abstract

**Background:**

Spinal dumbbell-shaped epidural cavernous malformation (CM) is a rare, hypervascular entity frequently misdiagnosed for other lesions, leading to unexpected intraoperative bleeding and suboptimal resection. Our study aims to elucidate the demographics, management strategy, and outcome of this vascular disease.

**Methods:**

Retrospective review of patients seen in Beijing Tiantan Hospital with symptomatic dumbbell-shaped epidural CM from 2008 to 2013. All lesions were pathologically confirmed after resection. The clinical manifestations, radiographic features, and treatment modalities of these cases were analyzed.

**Results:**

We included 9 consecutive patients. Average age was 58 ± 12 years (range: 34–79 years), with 66.7% male. Locations of the CMs were: thoracic (*n* = 7, 77.8%), cervical (*n* = 1, 11.1%), and cervicothoracic junction (*n* = 1, 11.1%). Only one case presented with acute manifestations while others experienced chronic progressive spinal cord symptoms. The initial clinical diagnoses were: schwannoma (*n* = 6, 66.7%), cavernous hemangioma (CM) (*n* = 1, 11.1%), meningioma (*n* = 1, 11.1%), and angioma (*n* = 1, 11.1%). Total resection was achieved in six patients (66.7%), and partial resection in the other three patients (33.3%). Average intraoperative blood loss was 400 ± 300 ml (range: 100–1000 ml). During an average follow-up of 71 ± 21 months (range: 29–94 months), excellent outcome was achieved in seven cases (77.8%), one partially improved (11.1%), and one deteriorated (11.1%). No patients experienced recurrence of symptoms.

**Conclusions:**

Spinal dumbbell-shaped epidural CM is a benign vascular malformation that should be differentiated from other dumbbell-shaped lesions. Accurate preoperative diagnose is challenging as no specific radiographic marker has been established. Total surgical resection should be recommended.

## Background

Spinal hemangiomas, or spinal cavernous malformations (CM), are a rare albeit benign vascular lesion that usually of intra-osseous origin with occasional extension into the epidural space. However, epidural CM without any bony involvement is rarely seen, and a dumbbell-shaped CM may be formed after extra-foraminal extensions under exceedingly rare conditions.

Owing to their scarcity, only 11 cases to our knowledge have been reported to date [1–9]. These lesions often morphologically mimic dumbbell-shaped tumors and purported high rate of misdiagnosis before surgical intervention [1]. It is imperative to realize that accurate diagnosis is critical for these types of lesions given that the surgical techniques and outcomes are distinct from other dumbbell-shaped neoplasms, and intraoperative hemorrhage and the clinical outcomes may be formidable considering their hypervascularity. This study is undertaken to analyze the radiological features, surgical management and evaluate the surgical outcomes of nine dumbbell-shaped epidural CM.

## Methods

A retrospective study was conducted after the consent of our Institutional Review Board (IRB) of Beijing Tiantan Hospital. Patients presenting with symptomatic spinal dumbbell-shaped epidural CM between June 2008 and September 2013 were included. The baseline data of the patients that including age, gender, clinical presentations, spinal radiological features, procedural characteristics, and surgical outcomes was electronically retrieved and reviewed.

When surgical intervention was considered after careful clinical evaluation and spinal cord magnetic resonance imaging (MRI) examination, the procedure was performed through a posterior approach in a lateral position under general anesthesia. The extent of resection was defined as total, subtotal, or partial. Total resection is defined as extirpation of both the intraspinal and extraforaminal portion of the lesion.

All the patients were routinely followed at the outpatient clinics for 3 and 6 months after discharge, and were assessed by telephone or outpatient visit every year subsequently. Follow-up images were obtained in all patients. Neurological outcomes were evaluated using the Modified McCormick Scale (MMS) [10]. A favorable functional outcome was defined as a MMS of 1 or 2 (neurologically intact or mild deficit), and a poor prognosis was defined as a MMS of 3, 4 or 5 (moderate deficit, severe disability or paraplegia).

## Results

### Baseline characteristics

Nine eligible patients were included with a mean age of 58 years (range: 34–79), with six (66.7%) male patients. Clinical course progression before presentation was mostly chronic except for one patient who presented with acute lower limb weakness after two years of chronic course (case two). Two cases were recurrent lesions, one of which underwent surgery four years ago and suffered a recurrent syndrome, and the other patient underwent two procedures at 14 years and 4 years ago, respectively. The preoperative presentations on admission were radiculopathy in 2 patients (22.2%), and myelopathy in 7 (77.8%) patients (manifesting as hypoesthesia, lower limb weakness, and urinary or bowel disturbances). The average duration of symptom onset was 11 months, with a range of 1–36 months. Preoperative MMS was as follows: two patients (22.2%) as MMS I, one (11.1%) as MMS II, four (44.5%) as type III, and two (22.2%) as type IV (Table 1).

**Table 1:** Characteristics of Nine Patients with Dumbbell-Shaped Epidural CM

	Age/Sex	Symptom	Duration(months)	Site	Initial Diagnosis	MRI findings			Radiologic features			Intra-op bleeding (ml)	Recurrent	FU/months	MMS	
						T1WI	T2WI	Gd	CT	Foramen Dilation	Extension				Pre-op	Last-FU
1	55/M	H/W/U	1	T2–4	Sch	Hypo	Hyper	HO/ob	Iso	Yes	D/Lt	200	No	94	III	I
2	58/M	H/W/B	24	T3–4	Sch	Iso	Hyper	HO/ob	Iso	No	D/Lt	200	No	94	IV	I
3	79/M	H/Lp	4	T7–8	CM	Iso	Hyper	HE/ob	Iso	No	Lt	200	Yes	94	II	I
4	66/M	H/W/U	6	T2–3	Sch	Iso	Hyper	HO/ob	Iso	No	D/Lt	1000	No	52	III	I
5	63/M	H/W/U	6	T3–4	Sch	Iso	Hyper	HO/ob	Iso	No	V/Rt	100	No	90	III	I
6	54/F	H/Lp	36	C7-T1	CM	Iso	Hyper	HO/ob	Iso	No	V/Rt	300	Yes	53	I	I
7	44/M	H/W	1	T7–9	Sch	Iso	Hyper	HO/ob	Iso	Yes	Rt	200	No	29	III	I
8	69/F	H/W/Cp	12	T5–6	Men	Iso	Hyper	HE/not ob	Iso	No	D/Rt	800	No	66	IV	III
9	34/F	Ap	12	C3–4	Sch	Iso	Hyper	HE/ob	Iso	Yes	Lt	600	No	67	I	II

*Abbreviations: Age/Sex: M* Male*, F:* Female*, Symptom: H:* Hypoesthesia*, W:* Leg Weakness*, U* Urinary retention*, B* Bowel disorders*, Lp* Leg pain*, Ap* Arm pain*, Cp* Chest pain*, Initial Diagnosis: CM* Cavernous malformation*, Sch* Schwannoma*, Men* Meningioma*, MRI: Iso:* Isointense*, Hype* Hyperintense*, Hyp* Hypointense*, HE* Heterogeneous-enhanced*, HO* Homogeneous-enhanced*,ob:* obvious*,D* Dorsal*,V* Ventral*,Rt* Right*, Lt* Left*; Pre-op* Preoperative*, FU* Follow-up

### MRI characteristics and preoperative diagnosis

**Fig. 1:**
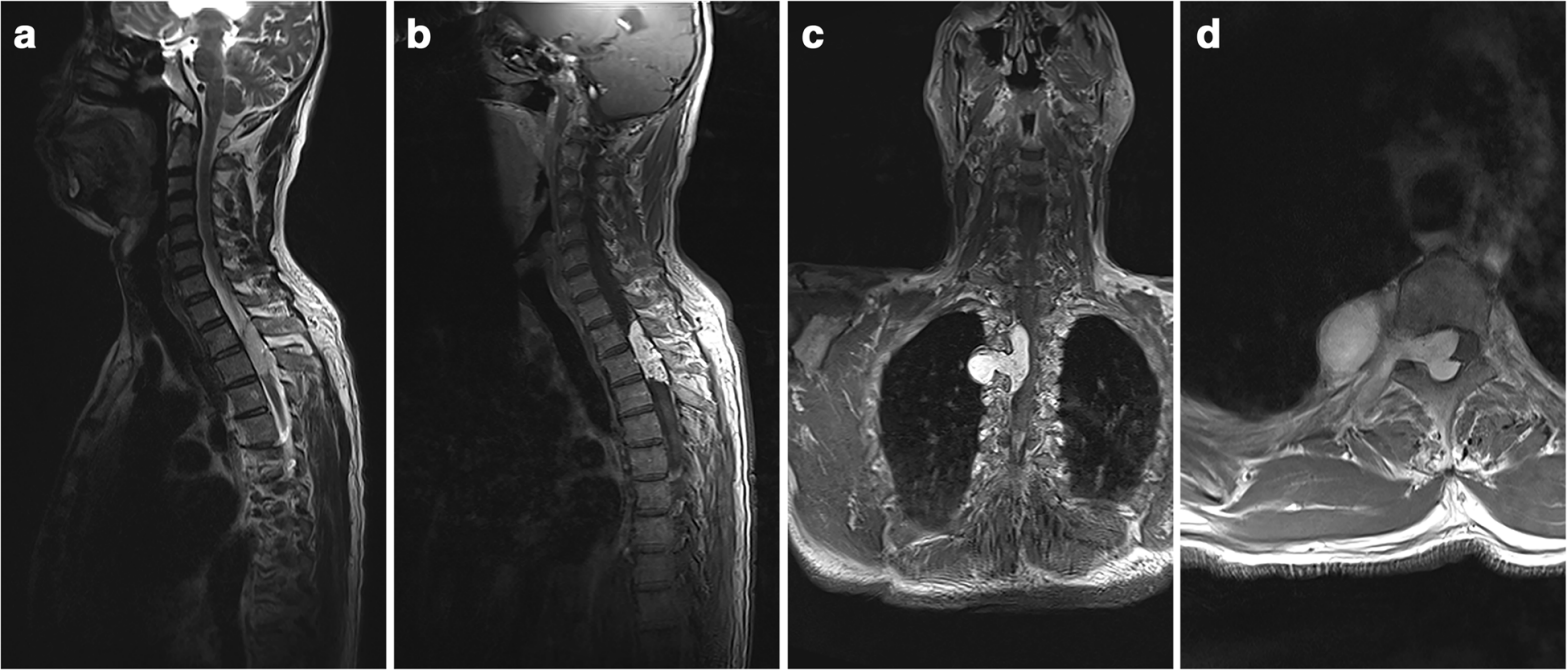
Preoperative sagittal T2-weighted magnetic resonance images (**a**) and contrast image (**b**, **c** and **d**) shows an epidural lesion from T2 to T3 level, extending through ipsilateral conjunction foramen and with intrathoracic extension (**b**, **c** and **d**). The epidural mass revealed isosignal intensity on T1-weight sequence and hypersignal intensity on T2-weighted sequence, with sagittal, axial and coronal view of homogeneous-enhanced after gadolinium injection. The lesions demonstrated isosignal intense in CT scan, without vertebral body erosion and enlargement of the intervertebral foramina

Spinal MRI demonstrated that the lesion location had a thoracic spine predominance (*n* = 7, 77.8%), followed by cervical (*n* = 1, 11.1%) and cervicothoracic junction (*n* = 1, 11.1%). The intraspinal part of the lesions were located in the posterior epidural space in four patients (44.6%), anterior space in 2 (22.2%) and lateral portion in 3 (33.3%). All lesions extended through the intervertebral foramen (5 lesions to the left side, 4 lesions to the right side) to present as an intrathoracic mass or cervical mass, which closely mimic the appearance of dumbbell-shaped tumors in coronal and axial view. All cases except for one exhibited isointense signal on T1-weighted images, with the exceptive case demonstrating hypointense signal. All cases had hyper-intensity on T2-weighted MRIs, and all lesions were enhanced after injection of Gadolinium (Fig. 1c). Three patients were revealed with dilated neuroforamen on MRI. On CT scans, all cases featured an iso-intense signal. Based on these preoperative materials, six lesions were radiologically misinterpreted as schwannomas, one as meningioma, and two as recurrent epidural hemangiomas.

### Surgical details intraoperative findings

All patients were symptomatic before surgery, and a posterior approach in a lateral position under general anesthesia was chosen [11, 12]. The preoperative decision was planned by conventional posterior approach without thoracoscope or assistance from the cardiothoracic team. Six patients (66.7%) underwent laminectomy, 1 hemilaminotomy (11.1%); the two recurrent cases (22.2%) already underwent previous removal of lamina. A lesion featured dark red or purplish red and well demarcated was revealed in the epidural space after exposure, and was found to be fragile, soft, and blood-rich in the process of dissection. Bipolar was utilized to coagulate the blood vessels and feeding arteries in the surface of the lesions and then shrink the lesions to reduce the hemorrhage volume and facilitate the process of resection. The intraspinal portion was found to be tightly attached to the dura mater; yet the margin of the lesion was easily detected and removed in an en bloc or piecemeal fashion. Notably, significant blood loss maybe encountered intraoperatively due to hypervascular content of the lesion, en bloc method is favored in dealing with intraspinal part for reducing bleeding, and bipolar for coagulation and tight Gelfoam sponge rolls were applied in the process of the resection to terminated bleeding. Two patients experienced extensive bleeding after resection, the bleeding was assumed to originate from the venous plexus around the tumor bed, and tight Gelfoam sponge rolls were used to suppress the cavity with satisfactory control of the bleeding despite rigorous hemostasis. Bone wax may also be used to prevent the hemorrhage when the tumor adhere to the bone or have bone erosion.

**Fig. 2:**
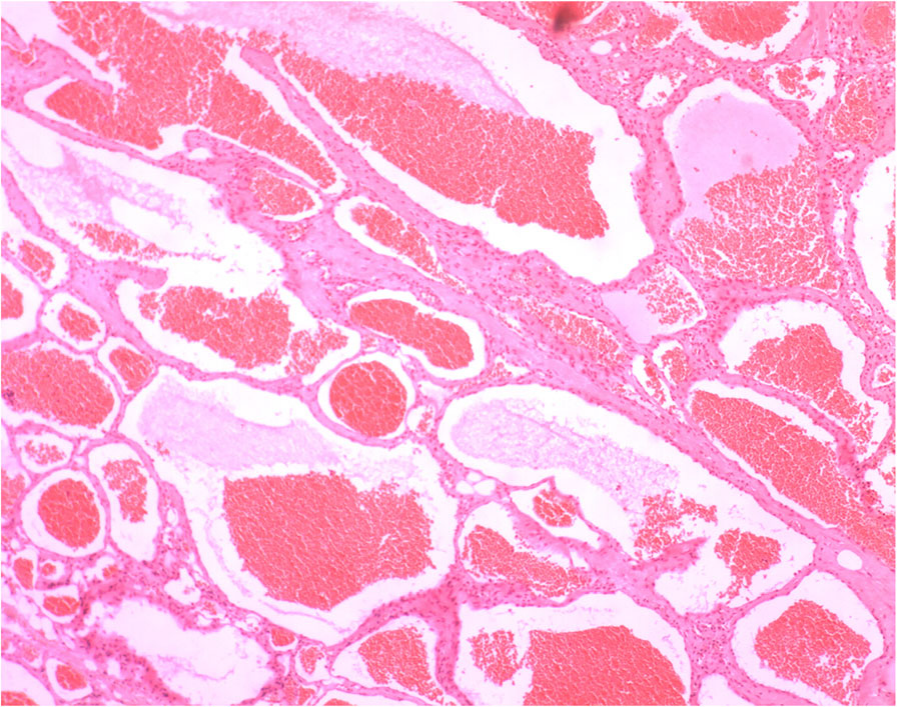
Photomicrograph examination demonstrating the diagnosis of cavernous hemangioma, showing the typical features of many irregular dilated blood-filled vessels lined with a single layer of endothelium. Some hemorrhage was observed in the vascular space. Hematoxylin and Eosin (H&E) staining, original magnification × 40

Following the total resection of the intraspinal portion, however, all lesions were discovered to have an extraforaminal extension. In five cases, the lesion extended out of the neuroforamen developing into a massive extraspinal portion; of these cases, this portion was subsequently resected through further exposure, and foraminotomy and/or partial medial facetectomy were performed without disrupting the osseous continuity of the interarticularis to avoid spinal stability. What’s more, costotransverse was sacrificed in one case to achieve total resection, the extraforaminal portion of the tumor can be detected and removable through the above exposure methods, whereas partial resection was achieved in another case in which the lesion was attached to the vertebral artery, for the remaining two cases only subtotal resection were achieved due to the limitation of the exposure in conventional approach. The tumors usually have a tight adhesion to the nerve root, but can be easily identified after resection of the intraspinal part and further exposure of the neuroforamen, and the nerve root can be easily separated from the tumor without injury. In the 2 recurrent cases, the lesions were found to be adhesive to the surrounding tissues without a clear border. Despite a challenging process, the lesion was completely removed. Average intraoperative blood loss for all patients was 400 ml. The pathological findings was showed in Fig. 2.

### Clinical outcomes and follow-up data

**Fig. 3:**
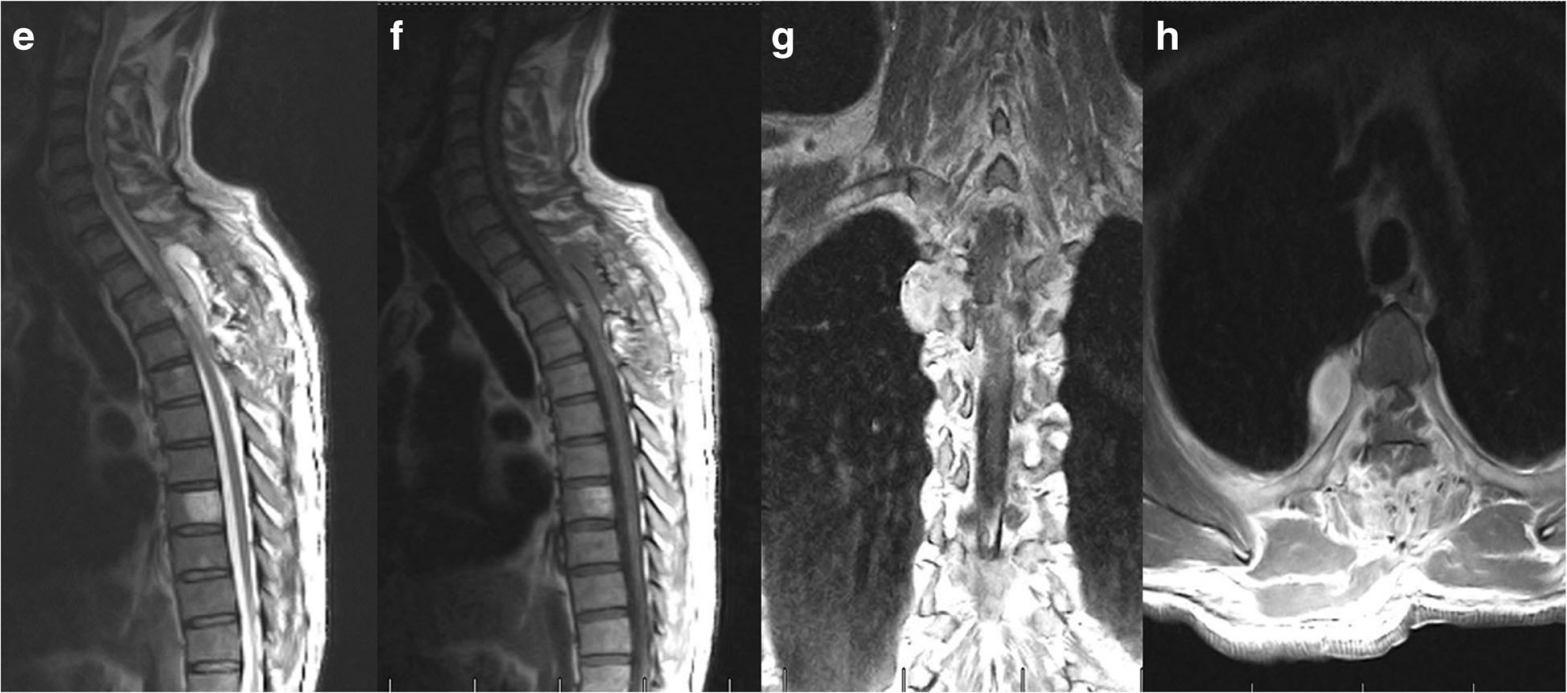
Postoperative sagittal T2-weighted magnetic resonance images (**e**) and contrast image (**f**, **g** and **h**) shows partial resection (the intraspinal part was totally removed and the extraforaminal portion was not resected)

No procedure-related complication occurred, and all patients had improvement to some degree immediately after operation. Postoperative MRI performed within 7 days and in follow-up period was carefully examined especially for residuals, and no hemorrhage or regrowth of the lesions was found (Fig. 3). Pain has completely resolved in patients presenting with pain, and symptoms such as weakness, sphincter disturbance, and sensory disorders significantly improved during an average follow-up period of 71 months (range: 29–94 months). However, one case remained functionally unchanged. Majority of these patients experienced no neurological or radiological progression; only one patient with partial removal of the lesion (case 9) experienced neurological progression of muscle atrophy of upper extremity.

## Discussion

Spinal cord CMs are considered to be rare vascular lesions with a congenital nature [9, 13]. According to classification on the basis of microscopic examination, they are categorized into four types (capillary, cavernous, arterio-venous, or venous), with cavernous type as the major pathological subtype and intramedullary as the most common location in the spinal cord. [1, 12] Pure spinal epidural CM accounts for only 4% of all spinal epidural tumors, and dumbbell-shaped subtype is even more scarce, with only 11 cases been reported in the form of case report [1, 2, 4, 6, 7, 9, 11, 14, 15].

According to published reports as summarized in Table 2, the median age of patients with spinal dumbbell CMs was 50 years, ranging from 23 to 77 years [1, 2]. Male to female ratio was 7:4 for the 11 reported patients, suggesting a potential predominance of male gender (63.6%, Table 2). Our cohort consisted of six male (66.7%) and three female patients with mean age of 44 years (range 27–56 years). These results are consistent with reported cases in the literature. The most common clinical presentation is myelopathic symptom and nerve root compression, occurring in 72.7% and 27.3%, respectively (Table 2). The duration of symptom onset varied from 1 month to 36 months with a median of 6 months [2, 4, 13, 14]. The compression to the spinal cord can account for myelopathy-related symptoms, whereas nerve root disturbance caused by lesion expansion to the neural foraminal and the mass effect of anterior part of the lesion may account for the symptom of pain [7, 13, 16, 17].

**Table 2:** Summary of Clinical Features and Epidemiological of 11 Reported Dumbbell-Shaped Epidural CM

Author	Age/Sex	Location	Presentation	Therapy	Resection	Intervetebral foramen	MRI features			FU
							T1WI	T2WI	Gd	
Rovira (1999)	51/F	L3–4	LBP, Sciatica	Hemilaminectomy	–	Not dilated	Iso	Hyper	HO	Improved
Franz (1987)	23/M	T3–4	Paraplegia	Laminectomy	Total	Dilated	–	–	–	Improved
Morioka (1986)	50/M	T2–3	Hypesthesia	Laminectomy + thoracotomy	Total	Not dilated	–	–	–	–
Haimes (1991)	46/F	T3–4	Hypesthesia	Laminectomy + thoracotomy	Total	Mild dilated	Iso	Hyper	–	Improved
Uchida (2010)	75/M	T11–12	Leg weakness, pain	Laminectomy + foraminotomy	Total	Not dilated	Iso	Hyper	HO	Improved
Lanotte 1994)	27/F	T1–2	Hypesthesia	Arthrolaminectomy	Total	Dialated	Hypo	Hyper	HO	Improved
Feider (1991)	50/M	L3–4	Sciatica	–	Total	Not dilated	Iso	Hyper	HO	Improved
Padovani (1982)	75/M	T3–6	Gait disturbance	Laminectomy	Partial	Not dilated	–	–	–	Improved
Fukushma (1987)	54/M	T7–8	Paraplegia	Laminectomy	Total	Not dilated	–	–	–	Improved
Harringto (1995)	37/F	L3–4	Leg numbness and pain	Hemilaminectomy	Subtotal	Not dilated	Iso	–	HE	Improved
Yunoki (2015)	77/F	L2–3	Hypesthesia	Foraminotomy	Subtotal	Mild dialated	Hypo	Hyper	HO	Improved

*Abbreviations*: *LBP*Low-back pain

Preoperative MRI is necessary for accurate diagnosis of spinal epidural CM [1, 9, 14, 18]. These lesions tend to display iso-signal or low signal on T1WI and high signal on T2WI, with homogeneous enhancement after injection of contrast material, most of which are located in the posterior epidural space and present with myelopathy [19, 20]. In previous reported cases with specified MRI findings (*n* = 7), six of which showed isosignal on T1WI and high signal on T2WI; additionally, four out of five with contrast material injection demonstrated homogeneous signal enhancement. On rare occasions, epidural CMs may be found to locate in the ventral extradural space and trigger radiculopathy, clinically and radiologically mimicking the presence of disk herniation.[13]On CT scans, these lesions appear as isodensity or slightly hyperdensity signals; however, they are less likely to have a dilated intervertebral foramina, with only 33.3% (*n* = 3) our series and 27.3% (*n* = 3) in previous report cases. [1, 2, 16] Thoracic segment is the predominant location as demonstrated with eight (72.7%) cases in the thoracic segment in the previous reports and seven (77.8%) in our series.

Dumbbell-shaped epidural CMs are almost indistinguishable from that of a neurinoma, neurilemmoma or meningiomas [13, 18, 21, 22]. Morioka et al. proposed to differentiate these tumors and spinal CM by enlargement of intervertebral foramina, suggesting that the latter is rarely likely to cause dilation compared to the former [6]. Conversely, this proposal is contradicted by Lee et al., who found that CM usually leads to a neural foraminal widening [18]. These lesions not only show dilated intervertebral foramina, but also tend to invade the vertebral body, and pedicle of vertebral arch, with heterogeneous enhancement and enhance less than spinal CMs and usually with cystic changes, which is different from spinal epidural CMs. Furthermore, spinal malignant tumors such as lymphoma, metastatic tumors, or sarcoma, which has been reported to constitute 10–40% of dumbbell-shaped spinal tumors, also tend to lack a dilated intervertebral foramina similar to that of spinal epidural CMs [13, 16, 23]. Therefore, provided with high potentiality of significant intraoperative bleeding, preoperative preparation of worst case-scenario is critical, and the diagnosis of spinal CM is recommended to be prioritized in the differential diagnosis of dumbbell-shaped lesions even when no dilation of intervertebral foramina is found. In our series, excluding the two recurrent cases, six cases were preoperatively diagnosed as schwannomas, with another case diagnosed as meningioma. This result and previous report suggests that the most common preoperative misinterpretation of dumbbell spinal epidural CM was schwannoma.

Complete resection is currently the best option for management of epidural dumbbell-shaped CMs in cases with high risk of rebleeding and progressive neurologic deterioration with acceptable outcome.[12, 14]However, for incompletely resected lesions, even no rebleeding and regrowth occurred in our series, the potential of event occurrence still remains, and radiation therapy has been recommended as an alternative. Sohn et al. observed no growth of lesion at 3-years follow-up after stereotactic radiosurgery for a residual lesion [24]. However, the effect of adjuvant radiosurgery remains to be proven since its long-term benefit is unclear [1, 24].

### Limitations of study

This study is a retrospective study and potential biases exist. Given the limited sample size, the conclusion inferred from the results of this single series might be biased and susceptible to random observation. However, this is still the largest series on spinal dumbbell-shaped epidural CM reported to-date.

## Conclusions

Spinal dumbbell-shaped epidural cavernous CM is a rare vascular entity with potential extensive intraoperative bleeding. It is therefore recommended that this pathology should be considered in the differential diagnoses of other paravertebral dumbbell-shaped lesions. According to our experience and reported cases surgical resection may achieve satisfactory outcomes with rigorous and meticulous drive of hemostasis. Although extraforaminal portion is commonly inaccessible and maybe left with residual lesions with conventional laminectomy, no recurrent bleeding or growth of the remaining lesion was observed in the long-term follow-up.

## Abbreviations

CM: cavernous malformation
IRB: Institutional Review Board
MMS: Modified McCormick Scale
MRI: magnetic resonance imaging;
